# Dietary L-citrulline supplementation modulates nitric oxide synthesis and anti-oxidant status of laying hens during summer season

**DOI:** 10.1186/s40104-020-00507-5

**Published:** 2020-10-12

**Authors:** Victoria A. Uyanga, Hongchao Jiao, Jingpeng Zhao, Xiaojuan Wang, Hai Lin

**Affiliations:** Department of Animal Science, College of Animal Science and Veterinary Medicine, Shandong Provincial Key Laboratory of Animal Biotechnology and Disease Control, Shandong Agricultural University, No. 61 Daizong Street, Tai’an, 271018 Shandong China

**Keywords:** Arginine, Antioxidants, Laying hens, L-citrulline, Nitric oxide, Summer

## Abstract

**Background:**

L-citrulline (L-Cit), a non-protein amino acid, has been implicated in several physiological functions including anti-inflammatory, anti-oxidative, and hypothermic roles, however, there is a paucity of information with regards to its potential in poultry production.

**Methods:**

This study was designed to investigate the effects of dietary L-Cit supplementation on the production performance, nitric oxide production, and antioxidant status of laying hens during summer period. Hy-Line Brown laying hens (*n* = 288, 34 weeks old) were allotted to four treatment, 6 replicates of 12 chickens each. Dietary treatments of control (basal diets), 0.25%, 0.50% and 1.00% L-Cit supplementation were fed to chickens for eight (8) weeks. Production performance, free amino acid profiles, nitric oxide production, and antioxidant properties were measured. Blood samples were collected at the 4^th^ and 8^th^ weeks of the experiment.

**Results:**

Air temperature monitoring indicated an average daily minimum and maximum temperatures of 25.02 °C and 31.01 °C respectively. Dietary supplementation with L-Cit did not influence (*P* > 0.05) the production performance, and rectal temperature of laying hens. Egg shape index was increased (*P* < 0.05) with increasing levels of L-Cit. Serum-free content of arginine, citrulline, ornithine, tryptophan, histidine, GABA, and cystathionine were elevated, but taurine declined with L-Cit diets. Plasma nitric oxide (NO_x_) concentration was highest at 1% L-Cit. Likewise, nitric oxide synthase (NOS) activity for total NOS (tNOS) and inducible NOS (iNOS) were upregulated with increasing L-Cit levels, although, tNOS was not affected at the 4^th^ week. Anti-oxidant enzymes including catalase and superoxide dismutase (SOD) were increased with L-Cit supplementation, however, SOD activity was unchanged at 4^th^ week, while total anti-oxidant capacity increased at the 8^th^ week. L-Cit supplementation attenuated the extent of lipid peroxidation, and also inhibited glutathione peroxidase activity.

**Conclusion:**

Dietary L-Cit supplementation modulated systemic arginine metabolism, nitric oxide synthesis, antioxidant defense system, and increased the egg shape index of laying hens during the summer season. 1% L-Cit supplementation proved most effective in potentiating these effects and may be adopted for feed formulation strategies.

## Background

In the tropics and sub-tropical regions, there are several constraints to poultry production with high environmental temperatures being the most obvious constraint. Summer seasons are typically characterized by temperature surges, since diurnal rise in temperature is often limited and subsequently followed with low ambient temperature. Several investigations have reported that summer heat adversely affects poultry production and welfare [1, 2], thus raising the need to understand the impacts and possible solutions for the alleviation of heat stress effects on livestock and poultry production [3–5]. Of interest, is the adoption of nutritional manipulations as a measure to ameliorate heat stress in poultry production. These interventions include adjustment of nutrient density, feed formulation to meet metabolic requirements, as well as, the inclusion of feed additives, such as vitamins, minerals, antioxidants, amino acids, probiotics, prebiotics, organic acids, essential oils and electrolytes [6–8]. These strategies are targeted at minimizing metabolic heat generation, improving water balance, electrolytes and nutrient intake, reduction in energy losses, and attenuating reactive oxidative species (ROS) production [3, 5, 9].

As a biological messenger, the role of nitric oxide (NO) in thermoregulation of mammals has been widely demonstrated and attributed to its actions on the sympathetic nervous system [10]. During thermal stress, NO as an endothelial vasodilator [11] acts to stimulate peripheral blood flow, thereby increasing autonomic heat dissipation. Likewise in chicks, NO has been shown to act via the central nervous system (CNS) to regulate body temperature [12]. Most biological functions of NO are mediated via the activities of the NO synthase (NOS), which includes, endothelial NOS (eNOS), neuronal NOS (nNOS) and the inducible NOS (iNOS) isoform [13]. Under normal conditions, eNOS and iNOS expression were observed in the heart tissues of broilers [14], and increased with heat exposure [15]. Also, heat stress-induced iNOS protein expression has been related to cardioprotective functions [16, 17]. These NOS enzymes actively catalyze the conversion of L-arginine to NO and L-citrulline (L-Cit) [13]. As an endogenous precursor for arginine synthesis, citrulline, a non-protein amino acid has been proposed to effectively restore arginine balance [18]. This stems from findings that citrulline can bypass hepatic catabolism and functionally recycle into arginine in systemic blood flow. Agarwal et al. [19] showed that dietary L-Cit supplementation in mice diets efficiently increased plasma arginine concentrations (35%) higher than arginine supplementation, since about 70% of supplemental arginine were subjected to splanchnic first-pass metabolism (FPM). Therefore, L-Cit ability to recycle into arginine, essentially augments NO-production in a dose-dependent manner [13, 20].

Citrulline, which can be obtained from *Citrullus vulgaris* (watermelon) is widely gaining research interest primarily due to its effects on muscle protein synthesis [21, 22], enterocyte functioning [23], as well as its indirect effects via arginine and NO production. Several studies highlight citrulline as a pharmaconutrient [18] as well as its broad therapeutic application in conditions including muscular dysfunctions [24], metabolic syndromes [25], endothelial dysfunction [26], and urea cycle disorders [27], etcetera. Also, L-Cit has been shown to exhibit antioxidant properties [28] mostly mediated via direct reduction of hydroxyl radical formation, or indirect actions of NO synthesis [29]. Very few studies have investigated the potentials of citrulline in chickens. It was observed that plasma citrulline concentration was reduced following exposure to heat stress conditions in chicks [30]. Furthermore, L-Cit has been ascertained to possess hypothermic functions in chickens following its ability to depress rectal temperature under ambient temperatures [31] and in heat-stressed chicks [32]. The utilization of watermelon rinds, as a rich source of citrulline, was shown to reduce rectal temperatures of chicks similarly as citrulline’s actions, under ambient and heat stress temperatures [33]. Also, as a potential feed additive, watermelon rind supplementation increased plasma citrulline levels in chicks [34]. These findings of L-Cit associated thermotolerance suggest the potential of L-Cit supplementation as a nutritional strategy that would assist in ameliorating summer heat stress. Therefore, this study was designed to investigate whether dietary L-citrulline supplementation could influence the production performance, nitric oxide production and, antioxidant status of laying hens reared during summer season.

## Methods

### Experimental animals and management

Two hundred and eighty-eight (288) Hy-Line Brown laying hens, at 34 weeks of age were used for this study. Laying hens were reared in a closed housing system, with forced ventilation and cage dimension of 60 cm × 46 cm × 44 cm. Each cage was equipped with a nipple drinker, and contained 3 birds. Groups of 4 cages represented 1 replicate of an experimental unit (12 birds per replicate). Hens were randomized to have similar average laying rates of 86% per replicate. Birds were allowed 1 week adaptation period and fed basal diets (corn-soybean meal-based diet containing 16.5% CP, 2700 ME kcal/kg, 3.5% calcium, and 0.4% available phosphorus) before the start of the experiment. Afterward, hens were allowed ad-libitum access to water and experimental diets for the 8 weeks study period. Daily lighting regime was maintained at 16 h light and 8 h of darkness.

### Experimental design and diets

Birds were arranged into 4 treatments, 6 replicates with 12 hens each. Dietary treatments were prepared by substituting L-citrulline powder (98%) for equal amounts of corn at quantities of 0, 0.25%, 0.5%, and 1.0% as shown in Table 1. L-Cit was purchased from Shandong Fosun Biotechnology Co., Ltd., China, and feed formulation was according to the National Research Council standard [35]. Experimental diets were prepared twice during the study period (4 weeks interval) to prevent feed deterioration and stored in a well-ventilated room. The experiment was conducted during the summer months of July and August.

**Table 1:** Calculated dietary composition and nutrient levels of experimental diets

	Control	0.25% L-Cit	0.50% L-Cit	1.00% L-Cit
Corn (8.5% CP)	54.055	53.805	53.555	53.055
Wheat bran	7.99	7.99	7.99	7.99
Soya bean oil	1.79	1.79	1.79	1.79
Soybean meal (43%)	24.29	24.29	24.29	24.29
Salt	0.35	0.35	0.35	0.35
Limestone	9.6	9.6	9.6	9.6
Dicalcium phosphate	1.44	1.44	1.44	1.44
Choline chloride (50%)	0.09	0.09	0.09	0.09
L- Lysine (99%)	0.1	0.1	0.1	0.1
DL- Methionine (99%)	0.17	0.17	0.17	0.17
Mineral premix ^a^ (0.1%)	0.1	0.1	0.1	0.1
Vitamin premix ^b^ (0.025%)	0.025	0.025	0.025	0.025
L-citrulline (98%)	0	0.25	0.5	1
Total	100	100	100	100
Nutrients levels				
Total CP, %	16.5	16.85	17.19	17.89
Non-citrulline CP, %	16.5	16.48	16.46	16.42
Metabolizable energy, kcal/kg	2700	2700	2700	2700
Calcium, %	3.5	3.5	3.5	3.5
Available phosphorus, %	0.404	0.404	0.404	0.404
Digestible lysine, %	0.78	0.78	0.78	0.78
Digestible methionine, %	0.4	0.4	0.4	0.4
Digestible methionine + cysteine, %	0.619	0.619	0.618	0.616
Digestible threonine, %	0.505	0.504	0.504	0.502
Digestible tryptophan, %	0.161	0.161	0.161	0.161

^a^ The mineral premix provide the follow quantities per kilogram of diet: iron, 55 mg; selenium, 0.3 mg; copper, 5.5 mg; zinc, 88 mg; I, 1.7 mg; manganese, 88 mg.

^b^ The vitamin premix provide the follow quantities per kilogram of diet: vitamin A, 8800 IU; vitamin D_3_, 3300 IU; vitamin K, 2.2 mg; vitamin E, 16.5 IU; cholecalciferol, 2800 IU; riboflavin 18.0 mg; niacin, 50 mg; pantothenic acid, 28 mg; biotin, 0.1 mg; folic acid, 0.6 mg

### Production performance

Egg production and egg weights were recorded daily, while body weights and feed intake were recorded weekly throughout the experimental period. Data computed were feed conversion ratio (FCR), laying rate, and egg mass. FCR was calculated as the ratio between feed intake and egg mass.

### Air and rectal temperature monitoring

Air temperature was monitored during the experimental period using temperature data recorder (ZDR-41, ZaDa, HangZhou, China) which were strategically wired on the cages to record from the 3 tier positions (top, middle and bottom). Air temperature logging was carried out for 6 weeks during the experimental period. Rectal thermometer was used to record cloacal temperatures from 2 hens per replicate weekly, by inserting probe 2–3 cm within rectum for 60 s.

### Egg quality assessment

In the 8^th^ week, random samples of 10 eggs per replicate (total of 240 eggs) were collected on a single day. Egg quality indices such as egg weights, yolk weights, and shell weights were measured using a sensitive weighing balance. Yolk color (1 to 15 based on the yolk color chart), yolk grade (AA, A, B or C), albumen height (± 0.1 mm), and Haugh unit (0–130) were measured using automatic egg multi tester (EMT-5200, Touhoku Rhythm Co. Ltd., Japan). Shell thickness was measured using an echometer (D-56 Wuppertal 1, Karl Deutsch), egg lengths, and egg width were measured using the vernier caliper. Egg shape index was calculated as egg breadth/egg length, and albumen weight was computed as the difference between egg weight, shell weight, and yolk weight.

### Blood sampling and analysis

Blood samples were collected twice during the experiment, at the 4^th^ and 8^th^ week, the hens were fasted for 12 h before sampling. Blood sample was collected from the wing vein using a heparinized syringe of 5 mL capacity, with 23 guage (0.6 mm × 25 mm) hypodermic needles. Plasma and serum samples were obtained after centrifugation at 3000×*g* for 10 min at 4 °C and stored at *−* 20 *°*C until further analysis.

### Plasma metabolites

Plasma concentrations of glucose, aspartate aminotransferase (AST), urate, and total protein were measured spectrophotometrically using Hitachi L-7020 automatic biochemical analyzer (Hitachi High-Technologies Corp., Tokyo, Japan). Plasma triglyceride (TG) and total cholesterol (TCH) were detected with test kits according to the manufacturer’s guidelines (Jiancheng Bioengineering Institute, Nanjing, Jiangsu, China). TG was determined via glycerol phosphate oxidase peroxidase assay, with repeatability (CV) ≤ 4.0% and inter-assay difference ≤ 6.0%. TCH assay was based on the enzyme-coupled reaction that quantifies both cholesterol esters and free cholesterol, with a repeatability (CV) ≤ 3.0% and inter-assay difference ≤ 5.0%. Calorimetric determination for TG and TCH was at 540 nm using a microplate reader (Elx808, Bio-Tek Winooski, VT).

### Amino acid (AA) profiling

Free AA concentration in serum was determined by ion-exchange chromatography using Hitachi L-8900 Amino acid Analyzer (HITACHI High-Tech Science, Japan). Serum sample (1 mL) was mixed with 40 mg of salicylic acid to allow deproteinization, then vortexed and stored at 4 °C for 10 to 12 h. Thereafter, samples were centrifuged at 4 °C, 12,000 × *g* for 30 min, and the supernatant was collected and passed through a filter (0.22 μm) before reading in the amino acid analyzer. The ratios of circulating amino acid metabolites associated with arginine metabolism were calculated [36].

### Determination of nitric oxide concentration

Nitric oxide actively metabolizes into nitrite (NO_2_^−^) and nitrate (NO_3_^−^) and the sum of both nitrate and nitrite concentrations (NO_2_^−^ + NO_3_^−^) represents the levels of nitric oxide (NO_x_) *in vivo*. In this study, the concentration of NO_2_^−^ was measured by color intensity after the reduction of NO_3_^−^ into NO_2_^−^ by nitrate reductase. NO_x_ concentration in plasma was detected using a commercial kit (Jiancheng Bioengineering Institute, Nanjing, Jiangsu, China). Supernatant was collected and absorbance was detected at 540 nm using a microplate reader (Elx808, Bio-Tek Winooski, VT).

### Detection of nitric oxide synthesis

Systemic nitric oxide synthase activity, that is, total NOS (tNOS) and inducible NOS (iNOS), were tested with commercial kits according to the manufacturer’s protocols (Jiancheng Bioengineering Institute, Nanjing, Jiangsu, CN). NOS catalyzes the reaction of L-arginine and molecular oxygen to produce NO. The NO formed reacts with nucleophilic substances to produce nonferrous compounds. The presence or absence of calcium was used to determine the calcium-dependent activity of tNOS and calcium-independent activity of iNOS [37]. The reaction’s absorbance was measured with 1 cm cuvets at 530 nm using a UV-2450 spectrophotometer (Beijing PGeneral, Beijing, China).

### Anti-oxidant parameters

Plasma antioxidant parameters including superoxide dismutase (SOD), total antioxidant capacity (T-AOC), glutathione peroxidase (GSH-Px), catalase (CAT) and malondialdehyde (MDA) were determined using commercial kits based on manufacturer’s guidelines (Jiancheng Bioengineering Institute, Nanjing, Jiangsu, China).

T-AOC was determined based on the ferric reducing antioxidant power assay (FRAP) principle where antioxidants reduce ferric tripyridyltriazine complex (Fe^3+^-TPTZ) to ferrous tripyridyltriazine (Fe^2+^-TPTZ) and the reaction absorbance was read at 570 nm. For SOD activity, samples were pre-tested to achieve 40–60% inhibition rates. The assay was performed with an intra-assay CV of 5.05% and inter-assay CV of 3.32% at 450 nm absorbance. CAT activity was determined using the visible light method where hydrogen peroxide (H_2_O_2_) is decomposed by CAT and ammonium molybdate is added to stop the reaction. The remaining H_2_O_2_ reacts with ammonium molybdate to form a complex which was determined at 405 nm and calculated as catalase activity. GSH-Px catalyzes the reaction of H_2_O_2_ with reduced glutathione to produce water. GSH-Px activity was determined by expressing the consumption of reduced glutathione in the reaction system at 412 nm. Pre-testing was carried out to determine the correct dilution ratio of 45–50% inhibition rate. Also, the extent of lipid peroxidation was tested using the thiobarbituric (TBARS) principle of malondialdehyde activity, absorbance was measured at 532 nm. The reaction absorbance for SOD, CAT, GSH-Px, MDA, and T-AOC were measured using a microplate reader (Elx808, Bio-Tek Winooski, VT).

### Statistical analysis

Data collected were analyzed with one-way ANOVA using Statistical Analysis Software (version 8e; SAS Institute, Cary, NC, United States). The data were presented as mean ± SEM. Differences between the means were evaluated using Duncan’s Multiple Range Test. Linear and quadratic regression were carried out using R Studio version 1.2.5042 [38]. Differences were considered significant at *P* < 0.05.

## Results

### Air and rectal temperature

**Fig. 1:**
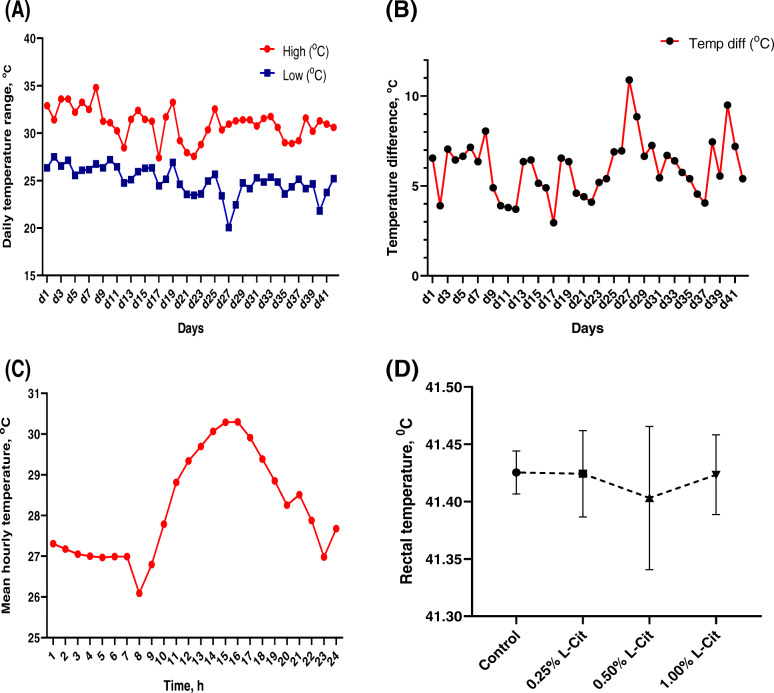
Air temperature data from hen house during the experimental period. (**a**) Mean daily maximum and minimum temperatures (**b**) Maximum and minimum temperature differences (**c**) Mean hourly temperatures during experimental period. (**d**) Rectal temperatures of laying hens

Daily air temperature recordings of the hen house is shown in Fig. 1. The diurnal range for maximum temperature was 27.4 –34.8 °C, average 31.01 °C, and the minimum temperature was 20.1–27.5 °C, average 25. 02 °C (Fig. 1a). Mean differences between the maximum and minimum daily temperatures ranged from 2.9 –10.9 °C (Fig. 1b). Hourly recordings of air temperature (Fig. 1c) showed that during the day, temperature started rising from 10:00 h, with an average peak of 30.3 °C between 14:00  to 15:00 h lasting for about 2 h before rescinding. Rectal temperature of laying hens (Fig. 1d) were not different (*P* > 0.05) between the 0.25%, 0.50% and 1.00% L-Cit groups and the control group. There were no significant changes between treatment groups during the weekly intervals (Fig. S1).

### Production performance and egg quality traits of laying hens

The effects of dietary L-Cit supplementation on the production performance of laying hens are shown in Table 2. Parameters measured were average daily feed intake (ADFI), body weight (BW), egg weight (EW), egg mass (EM), laying rate (LR), and feed conversion ratio (FCR). Hens fed with L-Cit diets at 0.25%, 0.5%, and 1.0% were not statistically (*P* > 0.05) different from the control group during the study period. The trend of changes observed with the laying rates, egg weights, and average daily feed intake during the study are provided in the additional files (Fig. S2 A-C).

**Table 2:** Effects of dietary citrulline supplementation on the production performance of laying hens from 32 to 42 weeks of age

	Control	0.25% L-Cit	0.50% L-Cit	1.00% L-Cit	*P*-value
ADFI, g/d	117.65 ± 2.99	115.68 ± 2.03	114.10 ± 2.76	114.31 ± 3.90	0.826
Final BW, kg/bird	1.88 ± 0.04	1.82 ± 0.05	1.89 ± 0.03	1.93 ± 0.01	0.938
EW, g	61.067 ± 0.39	61.43 ± 0.28	61.28 ± 0.83	61.68 ± 0.28	0.847
Egg mass, g/d	50.30 ± 1.90	50.71 ± 1.18	49.28 ± 2.09	48.23 ± 2.25	0.795
Laying rate	82.34 ± 2.88	82.57 ± 2.15	80.33 ± 2.72	78.20 ± 3.65	0.690
FCR	2.35 ± 0.05	2.29 ± 0.08	2.33 ± 0.080	2.39 ± 0.10	0.856

Data were presented as mean ± SEM, where *ADFI*Average daily feed intake, *BW* Body weight, *EW* Egg weights, *FCR* Feed conversion ratio (per g egg mass). Means with different superscripts within a row differ significantly (*P* < 0.05), (*n* = 12 chickens per replicate)

**Fig. 2:**
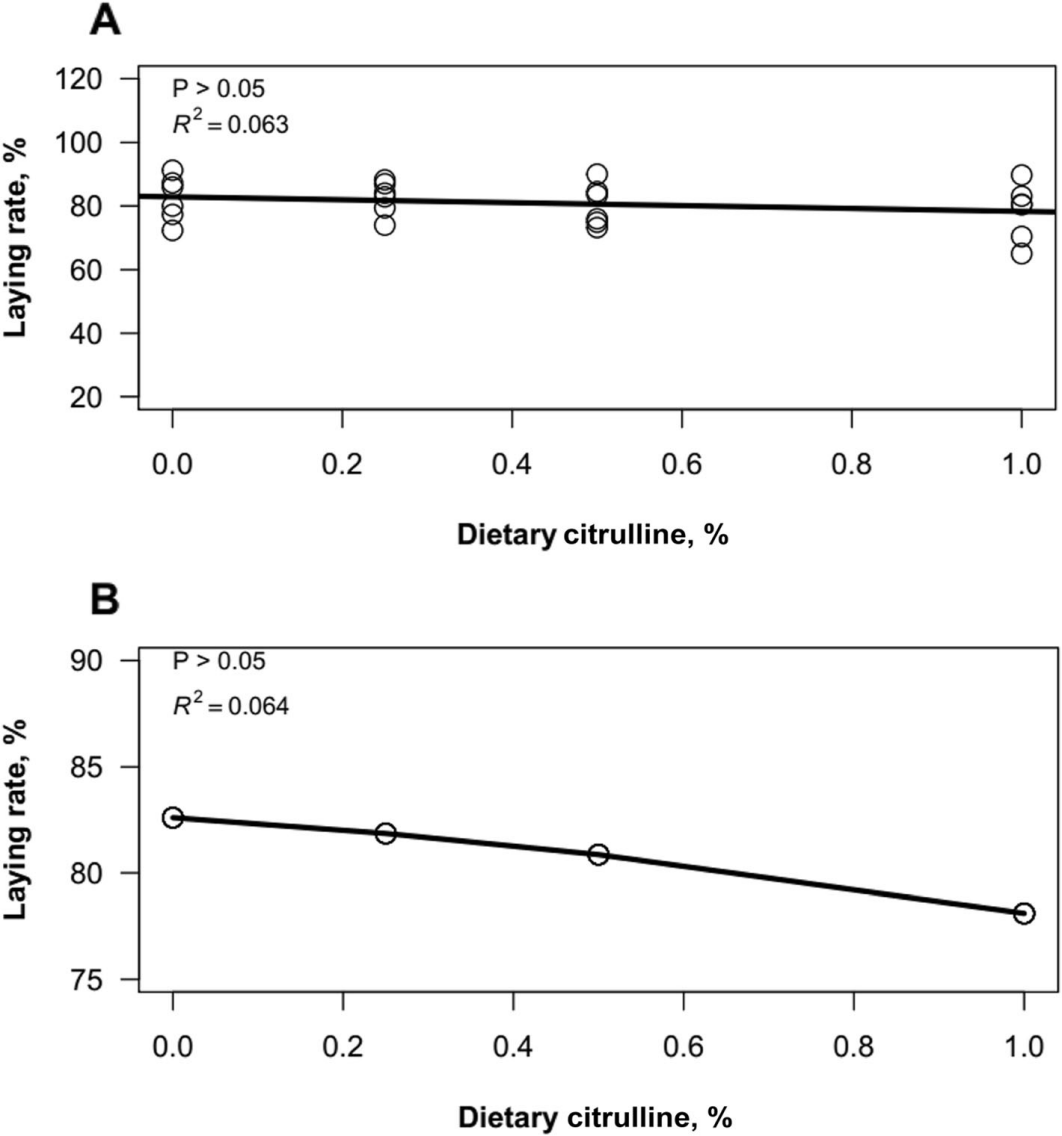
Regression model fitting for laying rate as a function of dietary citrulline levels in laying hens at 34–42 weeks old (**a**) Linear regression model (**b**) Quadratic regression model. Data are significantly different at *P* < 0.05

Egg quality traits were recorded as shown in Table 3. L-Cit supplementation significantly affected (*P* < 0.05) egg shape index. L-Cit fed hens had a higher shape index compared to the control group, although the shape index at 0.5% L-Cit was unchanged. Regression analysis was conducted to investigate the relationship between L-Cit levels and laying rate of hens (Fig. 2a, b). The scatterplot showed that there was a weak relationship between the two variables, and both linear and quadratic regression models depicted an insignificant relationship between L-Cit levels and laying rate (*P* > 0.05) (see additional files Table S1, S2).

**Table 3:** Effect of dietary citrulline supplementation on egg quality traits at 42 weeks of age

Parameters	Control	0.25% L-Cit	0.50% L-Cit	1.00% L-Cit	*P*-value
Shape index	0.74 ± 0.02^b^	0.77 ± 0.003^a^	0.77 ± 0.004^ab^	0.78 ± 0.003^a^	0.046
STK, mm	0.31 ± 0.005	0.31 ± 0.004	0.31 ± 0.003	0.31 ± 0.004	0.615
ESS, kg•f	3.45 ± 0.20	3.34 ± 0.17	3.54 ± 0.15	3.41 ± 0.15	0.851
AH, mm	6.19 ± 0.50	5.03 ± 0.31	5.69 ± 0.38	6.46 ± 0.47	0.066
Yolk color	5.31 ± 0.49	4.97 ± 0.46	5.51 ± 0.60	5.03 ± 0.55	0.869
Haugh unit	73.91 ± 3.58	65.76 ± 2.89	69.26 ± 2.89	75.53 ± 2.69	0.094
Yolk grade	3.22 ± 0.19	3.11 ± 0.16	3.15 ± 0.15	3.39 ± 0.12	0.625
Absolute YW, g	16.00 ± 0.19	16.00 ± 0.28	15.64 ± 0.26	16.03 ± 0.20	0.592
YW, %	26.26 ± 0.33	26.13 ± 0.44	25.62 ± 0.42	26.20 ± 0.36	0.637
Absolute SW, g	5.75 ± 0.11	5.88 ± 0.11	5.74 ± 0.10	5.64 ± 0.10	0.667
SW,%	9.50 ± 0.16	9.50 ± 0.16	9.38 ± 0.11	9.21 ± 0.13	0.457
Absolute AW, g	39.41 ± 0.82	39.48 ± 0.51	39.80 ± 0.63	39.70 ± 0.82	0.970
AW,%	64.33 ± 0.35	64.36 ± 0.46	64.99 ± 0.44	64.59 ± 0.37	0.618

Data were presented as mean ± SEM. ^a, b^: Means with different superscripts within the same row differ significantly at *P* < 0.05, (*n* = 240 eggs). *STK* Shell thickness, *ESS* Eggshell strength, *AH* Albumen height, *YW* Yolk weight, *SW* Shell weight, *AW* Albumen weight

### Biochemistry of plasma metabolites

The results on plasma metabolites including triglyceride, total cholesterol, glucose, total protein, uric acid, and aspartate aminotransferase are presented in Table 4. In the 8^th^ week, plasma TG content was significantly (*P* < 0.05) decreased with L-Cit diets compared to the control group. Other plasma metabolites at both 4^th^ and 8^th^ weeks were not affected (*P* > 0.05) among the control and L-Cit diet groups.

**Table 4:** Effect of dietary citrulline supplementation on plasma metabolites of laying hens at 32 and 42 weeks of age

	Control	0.25% L-Cit	0.50% L-Cit	1.00% L-Cit	*P*-value
4^th^ week					
TG, mmol/ L	2.25 ± 0.32	1.58 ± 0.33	2.14 ± 0.39	2.18 ± 0.44	0.570
TCHO, mmol/ L	6.64 ± 0.65	7.11 ± 0.80	5.86 ± 0.60	6.16 ± 0.30	0.510
GLU, mmol/ L	12.46 ± 0.41	12.12 ± 0.32	12.07 ± 0.34	12.54 ± 0.54	0.802
TP, g/L	48.33 ± 2.78	43.77 ± 2.73	53.38 ± 7.83	47.88 ± 7.00	0.690
UA, μmol/ L	169.83 ± 26.66	103.00 ± 21.25	108.17 ± 11.13	126.50 ± 36.50	0.270
AST, U/L	198.67 ± 20.81	213.17 ± 15.85	176.50 ± 11.17	185.50 ± 6.67	0.336
8^th^ week					
TG, mmol/ L	2.85 ± 0.28^a^	1.87 ± 0.24^b^	2.67 ± 0.31^ab^	1.94 ± 0.31^b^	0.045
TCHO, mmol/ L	4.14 ± 0.40	3.59 ± 0.33	4.39 ± 0.30	4.08 ± 0.29	0.401
GLU, mmol/ L	12.34 ± 0.35	12.39 ± 0.27	11.82 ± 0.63	12.40 ± 0.38	0.741
TP, g/L	49.20 ± 4.59	47.15 ± 1.63	41.20 ± 2.97	44.00 ± 2.39	0.296
UA, μmol/ L	151.75 ± 19.91	156.50 ± 24.15	179.00 ± 9.54	153.88 ± 22.05	0.747
AST, U/L	155.50 ± 13.58	148.63 ± 6.46	156.75 ± 11.68	166.63 ± 11.04	0.716

Data were presented as mean ± SEM, where *TG* Triglyceride, *TCHO* Total Cholesterol, *GLU* Glucose, *TP* Total protein, *UA* Uric acid and *AST* Aspartate aminotransferase. Means with different superscripts within a row differ significantly (*P* < 0.05) (*n* = 8)

### Serum-free amino acid content

**Fig. 3:**
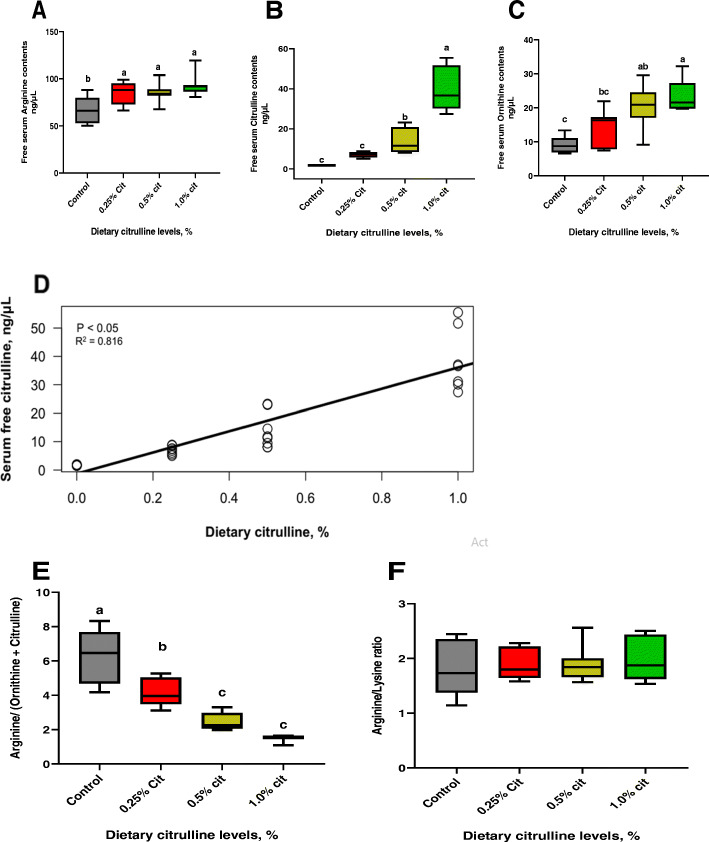
Free serum content of amino acids involved in Arginine metabolism. (**a**) Arginine (**b**) Citrulline (**c**) Ornithine (**d**) Serum free citrulline response to supplemental L-citrulline (**e**) Arg/Orn + Cit ratio (**f**) Arg/Lys ratio. Data are significantly different at *P* < 0.05; *n* = 8

The changes in serum-free amino acids of laying hens at 42 weeks of age are shown in Table 5. Essential amino acids such as tryptophan and histidine were significantly (*P* < 0.05) increased with higher L-Cit levels compared to control diets. Also, non-essential amino acids (NEAA) including GABA, and cystathionine were significantly (*P* < 0.05) increased, whereas, taurine was decreased (*P* < 0.05). Figure 3 shows the changes in serum-free contents for arginine (Arg), citrulline (Cit), and ornithine (Orn) as major amino acids involved in arginine metabolism. Their concentrations were significantly (*P* < 0.05) increased with higher levels of dietary L-Cit compared to the control group. Regression analysis showed that serum-free citrulline increased linearly (*P* < 0.05; *R*^2^ = 0.861) as supplemental L-Cit increased (Fig. 3d). Computed markers for arginine metabolism showed that the Arg to Orn + Cit ratio was declined (*P* < 0.05) systematically with increasing levels of L-Cit inclusion (Fig. 3e). The trend of changes depicted an inverse progression compared with the stepwise increment in serum L-Cit content following higher L-Cit levels. The Arg: Lys ratio was not significantly different (*P* > 0.05) among all treatment groups (Fig. 3f).

**Table 5:** Free amino acid contents in serum of laying hens fed dietary citrulline at 42 weeks old

Amino acids, ng/μL	Control	0.25% L-Cit	0.50% L-Cit	1.00% L-Cit	*P*-value
Essential amino acids					
Phenylalanine	21.02 ± 1.05	21.84 ± 0.96	21.79 ± 0.85	22.24 ± 0.75	0.824
Threonine	28.96 ± 2.07	35.04 ± 4.06	30.15 ± 5.44	36.73 ± 3.54	0.456
Valine	19.43 ± 0.73	23.36 ± 2.32	23.23 ± 2.14	24.53 ± 1.79	0.255
Methionine	15.38 ± 1.14	15.69 ± 0.77	15.57 ± 0.80	16.19 ± 1.01	0.942
Tryptophan	5.42 ± 0.30^b^	4.90 ± 0.26^b^	5.28 ± 0.53^b^	7.67 ± 0.88^a^	0.008
Lysine	42.15 ± 3.90	45.88 ± 3.29	43.27 ± 1.27	46.97 ± 3.43	0.681
Histidine	17.64 ± 1.09^c^	19.36 ± 0.96^bc^	22.53 ± 0.80^a^	20.78 ± 1.20^ab^	0.016
Isoleucine	11.43 ± 0.61	13.34 ± 1.22	13.45 ± 0.93	13.84 ± 0.68	0.302
Leucine	24.70 ± 1.04	27.61 ± 2.16	27.23 ± 1.50	26.20 ± 1.61	0.727
Non-essential amino acids					
Taurine	24.31 ± 0.73^a^	18.20 ± 0.78^b^	17.57 ± 1.26^b^	19.41 ± 2.08^b^	0.006
Aspartate	13.42 ± 1.48	16.19 ± 2.03	15.58 ± 1.44	16.53 ± 1.01	0.491
Serine	59.65 ± 4.72	63.90 ± 2.80	66.91 ± 4.00	66.53 ± 3.51	0.524
Glutamate	30.12 ± 2.51	29.11 ± 1.13	28.34 ± 0.91	26.91 ± 0.95	0.505
Glycine	42.74 ± 2.50	39.78 ± 2.39	44.44 ± 3.09	40.04 ± 2.07	0.518
Alanine	39.72 ± 2.50	42.18 ± 3.20	37.42 ± 1.62	40.14 ± 2.34	0.609
Cysteine	5.48 ± 0.80	4.46 ± 0.67	5.61 ± 0.99	5.09 ± 0.78	0.755
Tyrosine	25.87 ± 1.42	27.86 ± 2.22	27.19 ± 1.62	25.91 ± 0.85	0.771
β-Alanine	4.90 ± 0.51	4.51 ± 0.14	4.34 ± 0.46	5.65 ± 0.35	0.111
GABA	0.24 ± 0.02^b^	0.26 ± 0.02^b^	0.28 ± 0.03^b^	0.57 ± 0.06^a^	0.004
3-Methylhistidine	2.07 ± 0.15	1.72 ± 0.07	2.09 ± 0.13	1.54 ± 0.26	0.073
Cystathionine	1.05 ± 0.08^ab^	0.85 ± 0.04^b^	1.15 ± 0.08^a^	1.08 ± 0.07^a^	0.032
P-Serine	7.20 ± 0.59	6.53 ± 0.78	5.53 ± 0.3	5.88 ± 0.55	0.217
Sarcosine	2.06 ± 0.16	2.31 ± 0.08	2.24 ± 0.17	2.03 ± 0.20	0.556
Carnitine	2.46 ± 0.26	2.08 ± 0.19	2.20 ± 0.13	1.94 ± 0.11	0.241

Data were presented as mean ± SEM. Means with different superscripts within the same row differ significantly, *P* < 0.05 (*n* = 8); *GABA* means γ-aminobutyric acid

### Nitric oxide synthesis and production

**Fig. 4:**
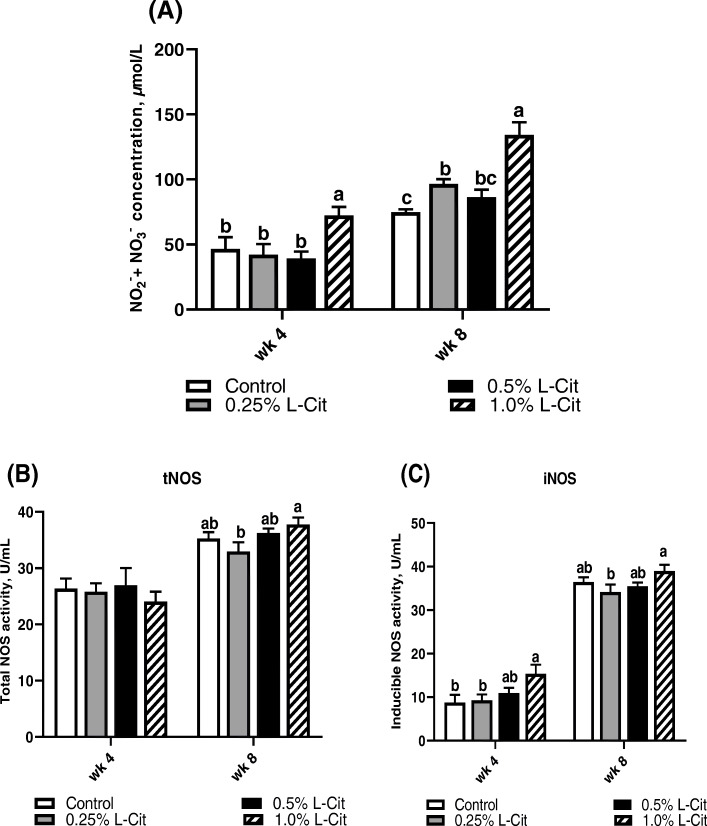
Effect of different L-supplementation levels on nitric oxide (NO_x_) production (**a**) Plasma NO_x_ concentrations; (**b**) Total nitric oxide synthase (tNOS) enzyme activity and (**c**) Inducible nitric oxide synthase (iNOS) enzyme activity. Values are means and SEM represented by vertical bars. Means with different superscripts differ significantly (*P* < 0.05) (*n* = 8)

Figure 4a shows the concentration of nitric oxide metabolites present in the plasma at different time points. At the 4^th^ and 8^th^ week, 1% L-Cit group had the highest (*P* < 0.05) NO_x_ concentration compared to other treatments. An increase of 36.7%, 56.4%, 54.5%, and 46.1% were observed between the 4^th^ and 8^th^ weeks for control, 0.25%, 0.5%, and 1.0% L-Cit groups respectively. The NO_x_ concentration for control, 0.25% and 0.5% L-Cit diets were not significantly different (*P* > 0.05) at the 4^th^ week, but 0.25% L-Cit increased significantly (*P* < 0.05) by 22.5% compared to control group at the 8^th^ week.

Plasma tNOS activity (Fig. 4b) was not significantly influenced by L-Cit treatment at 4^th^ week. In the 8^th^ week, 1% L-Cit group had higher tNOS activity compared to 0.25% L-Cit, but was not different (*P* > 0.05) compared with control and 0.5% L-Cit groups. The effects of L-Cit supplementation on iNOS enzyme activity is shown in Fig. 4c. At the 4^th^ week, 1% L-Cit diets had higher (*P* < 0.05) iNOS activity compared with control and 0.25% L-Cit diets but were not statistically different (*P* > 0.05) from the 0.5% L-Cit diet. However, in the 8^th^ week, iNOS activity for 1% L-Cit was significantly higher than 0.25% L-Cit. The iNOS activity from the 4^th^ to 8^th^ week showed an increase of 76.1%, 72.9%, 69.2% and 60.6% for control, 0.25%, 0.5%, and 1.0% L-Cit groups respectively.

### Antioxidant status

The effect of L-Cit supplementation on anti-oxidants parameters is shown in Fig. 5. During the 4^th^ week, plasma MDA content (Fig. 5a), was declined (*P* < 0.05) in a dose-dependent manner between the control group, 0.25% L-Cit and 0.5% L-Cit groups. However, this effect was abolished with 1% L-Cit group which had higher (*P* < 0.05) MDA contents compared to 0.5% L-Cit group. A similar decline in MDA content was observed at the 8^th^ week, as the control group had higher MDA contents although this was not significant (*P* > 0.05). SOD enzyme activity was not significantly (*P* > 0.05) influenced at the 4^th^ week (Fig. 5b), but, in the 8_th_ week, 1% L-Cit group had higher (*P* < 0.05) SOD activity compared with the control and 0.25% L-Cit group. Similarly, 0.5% L-Cit significantly (*P* < 0.05) enhanced SOD activity compared to 0.25% L-Cit diet.

**Figure:**
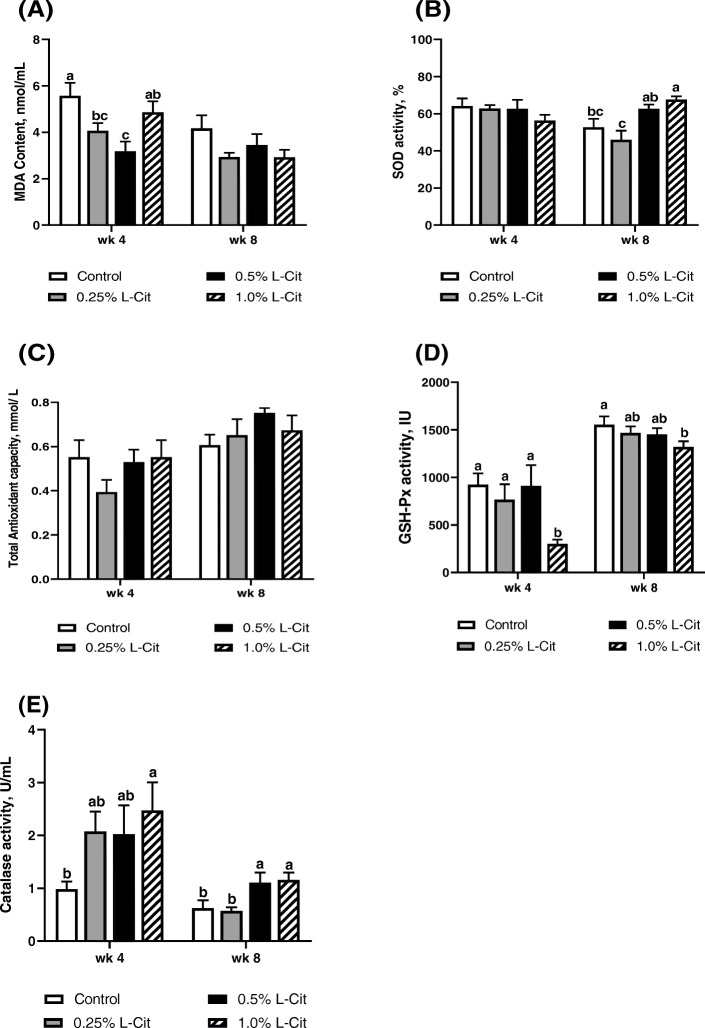
**Fig. 5** Effect of different L-supplementation levels on anti-oxidant properties of laying hens (**a**) Malondialdehyde (MDA) contents (**b**) Superoxide dismutase (SOD) activity (**c**) Total anti-oxidant capacity (T-AOC) (**d**) Glutathione peroxidase (GSH-Px) activity (**e**) Catalase (CAT) activity. Values are means and SEM represented by vertical bars. Means with different superscripts differ significantly (*P* < 0.05) (*n* = 8)

T-AOC capacity was not significantly different (*P* > 0.05) between treatment groups both at the 4^th^ and 8^th^ weeks (Fig. 5c). Plasma GSH-Px activity was observed to decline (*P* < 0.05) with 1% L-Cit diet (Fig. 5d) both at the 4^th^ week and 8^th^ week, however, the GSH-Px activity was similar (*P* > 0.05) for control, 0.25%, and 0.5% L-Cit diets. CAT activity was increased with L-Cit supplementation (Fig. 5e), such that, at the 4^th^ week, 1% L-Cit had significantly higher (*P* < 0.05) CAT activity compared with the control group. At the 8^th^ week, CAT activity was increased for both 0.5% and 1% L-Cit diets compared with control and 0.25% L-Cit groups (*P* > 0.05).

## Discussion

### Temperature monitoring and production performance of laying hens

Although several factors including air temperature, relative humidity, wind velocity, and previous acclimatization [39, 40] influences bird’s responses to temperature fluctuations, indoor temperatures above 30 °C have been associated with heat stress in laying hens [40]. This study focused on the influence of ambient temperature changes encountered during summer season on laying hens performance.

A relatively wide temperature range between 10 °C to 27 °C can allow for optimal growth whereas the highest feed efficiency is achieved at 27 °C [41]. Similarly, Abbas et al. [42] reported that layers housed within 14-28 °C had improved feed conversion, body weight, and body weight gains. Our findings indicate that during the summer study period, average minimum and maximum indoor temperatures of 25.02 °C and 31.01 °C exposed the birds to ambient conditions beyond thermal comforts. However, throughout the study period, we recorded no significant changes in the rectal temperatures of laying hens. Likewise, our findings indicate that L-Cit supplementation did not affect the growth and production performance of laying hens. The ADFI, BW, egg weight, egg mass, laying rate, and FCR were unchanged. Variations in solar intensity and duration of heated periods during summer may account for the absence or insignificant effects recorded on the performance of laying hens. Constant temperatures result in higher reductions in egg-laying performance compared to diurnal fluctuating temperatures [43]. This suggests that during the study period, the severity of heat exposure was not sufficient to initiate adverse effects on laying hens performance. Al-Saffar and Rose [43] reported that hens were better able to tolerate an increase in diurnal temperatures without marked effects on their productive performance. Our findings differ from Chowdhury et al. [31] who reported that oral administration of L-Cit reduced rectal temperature in chicks. Also, Kvidera et al. [44] showed that citrulline supplementation had a tendency (*P* = 0.07) to decrease rectal temperature but did not affect the production variables of pigs under thermal stress. These contrasting reports may be attributed to differences in the route of administration and heat stress conditioning. Hy-Line Brown commercial layers recommendations for ADFI (18–90 weeks; 105–112 g/d per bird), and FCR, kg feed/kg eggs (20–90 weeks; 1.95–2.07) were similar with our findings of 117.65 g/d ADFI and 2.35 FCR, although the laying rate differed from the expected standard of 95–96% [45]. This however could not be attributed to treatment conditions as the results were not significantly affected across all groups. Similarly, a laying rate of 74.74% at 40 to 46 weeks [46] and 86.2% from 21 to 35 weeks of age, had been reported from previous studies [47].

### Egg quality characteristics

The egg weight, shell thickness, eggshell strength, yolk color, yolk grade, yolk weight, albumen weight, and shell weight were unchanged in this study. These findings corroborate with Kilic and Simsek [40], who showed that during summer, the egg quality characteristics of laying hens were unchanged. Egg shape index depicts the proportion of egg breadth to egg length, as such, a vital tool in egg grading [48], and an important determinant of egg quality [49]. Our results showed that dietary L-Cit increased egg shape index, suggesting an improvement to the external egg quality. There exists a quantitative relationship between calcium concentration and citrulline synthesis in the mitochondrial matrix [50]. Liu et al. [46] reported that increased circulating free calcium (Ca^2+^) in the blood in combination with other plasma proteins involved with eggshell formation improved eggshell quality. This implies that L-Cit may directly/indirectly act to increase intracellular Ca^2+^ responsible for improving the egg shape index. This coincides with Sahin et al. [51] who demonstrated that enhanced calcium metabolism resulted in improved eggshell quality.

However, there were no observed changes in other measures of eggshell quality such as shell thickness, shell weights, and eggshell strengths. It had been reported that shell weights, breaking strengths and shell thickness were not affected by changes in egg shape index and there existed no significant correlation between these parameters [49, 52]. This disagrees with the report that shell thickness increased with increasing shape index [53]. These controversial findings provide opportunities for further research. Therefore, with relation to poultry feeding, this study demonstrates that citrulline supplementation in laying hen diets would not result in detrimental effects on egg production and egg quality. However, further studies with a longer experimental period are necessary to validate these findings.

### Plasma metabolites and circulating amino acid contents

In this study, the addition of L-Cit to the diet of laying hens did not alter several biochemical parameters of the birds. However, L-Cit mediated reduction in plasma TG may be linked to its direct and/or indirect effects on lipid metabolism. Previous studies have shown that dietary L-Arg reduced plasma TG and TCH contents, which was related to its downregulation of hepatic lipogenic enzymes [54]. Also, NO as a potent metabolite of Arg and Cit actions is implicated in the modulation of body fat deposits and fat metabolism [55]. Contrarily, Chowdhury et al. [32] observed that orally administered L-Cit did not affect plasma TG and TCH in chicks. From this study, the insignificant differences in plasma aspartate aminotransferase levels, an indicator of liver and kidney functions [56, 57], indicates that L-Cit feeding did not alter hepatic and renal functioning of laying hens.

Dietary L-Cit influenced the serum amino acid flux by increasing the availability of tryptophan, histidine, GABA, and cystathionine in systemic blood flow. Major amino acids involved in arginine metabolism including arginine, citrulline, and ornithine, were increased with L-Cit supplementation. Human and murine studies have shown that supplemental citrulline can efficiently increase systemic arginine, citrulline, and ornithine availability [13, 19, 58], however, our study demonstrates this finding in poultry. The indifference in Arg: Lys ratio, an index of Arg availability [36], suggest that citrulline inclusion in poultry diet would not result in antagonisms arising from arginine-lysine competition [59], as such, arginine catabolism due to high lysine intakes may be alleviated via citrulline inclusion. Furthermore, laying hens fed L-Cit diets displayed increased serum Arg, while Cit and Orn concentrations peaked with higher L-Cit levels. However, L-Cit modulation of these metabolites resulted in a diminished Arg to Orn and Cit levels, a measure of global arginine metabolism and bioavailability [25, 36]. This index captures the inter-organ recycling necessary to ensure systemic arginine supply [25]. Therefore, L-Cit modulation of systemic arginine concentration may not necessarily correspond to increased arginine bioavailability. This may be explained by the increased NOS activity and consequent elevation in systemic NO_x_ levels, suggesting an increased capacity for Arg catabolism, alongside Cit-Arg- recycling. In a fructose-induced model of nonalcoholic fatty liver disease (NAFLD), supplemented citrulline normalized AA fluxes and Arg metabolism, by restoring the plasma Arg-to-Lys ratios and Arg-to-Orn + Cit ratios [36].

### Systemic NO production

Studies in porcine and human models have reported L-Cit as a potent precursor for NO synthesis [24, 60], however, this is yet to be ascertained in poultry. Our study hypothesized that L-Cit, as an endogenous precursor of arginine, would modulate NO synthesis when supplemented in laying hen diets. Since Arginine biosynthesis in poultry is limited due to lack of carbamoyl phosphate synthase [61], and the several precursory roles of arginine including protein synthesis, urea cycle, production of polyamines, creatine and nitric oxide, may affect its priorities of use and bioavailability [61], the ability of supplemental L-Cit to facilitate the Arg- Cit- Arg recycling for NO synthesis will be of significant impacts for both arginine and NO-mediated functions [62, 63]. Earlier investigation on citrulline utilization in poultry reported by Klose and Almquist [64] demonstrated that citrulline supplementation to an arginine deficient diet effectively improved chick growth in a similar response as arginine addition.

Our study revealed that dietary L-Cit was able to increase systemic NO_x_, as well as the activities of NO synthases in a dose-specific manner with the highest levels (1% L-Cit) producing greater NO_x_ concentrations and NOS activities. NO_x_ concentration is positively associated with citrulline levels [65]. This association was demonstrated in our study by the increment observed between L-Cit levels and NO production since the quantitative assessment of plasma NO concentrations (NO_2_^−^ + NO_3_^−^) depicts the systemic NO synthesis [66]. The peak in plasma NO_x_ can be attributed to the generation of NO by NOS since both total NOS and inducible NOS activity were upregulated in the 8^th^ week. Several factors are involved in the regulation of iNOS expression including stress signals and inflammatory cytokines, however, iNOS expression has been implicated in both stimulatory and inhibitory actions [67]. Thus, aside from its role in NO synthesis, further research is necessary to ascertain the implications of L-Cit induced iNOS expression. Similar to our findings, Ham et al. [24] demonstrated that L-Cit induced iNOS mRNA expression was associated with increased mRNA expressions of endogenous antioxidants such as SOD1, SOD3, and catalase. Also, Kim et al. [68] showed that citrulline ingestion improved NO synthesis rate. These findings differ from Chowdhury et al. [32] who reported that oral injections of L-Cit did not significantly increase plasma NO_x_ concentrations compared with control group. The differences in reports may be attributed to the ages of birds, breed, and route of citrulline administration.

### Anti-oxidant status

Antioxidant enzyme (CAT, GSH-Px, and SOD) activities are typically upregulated after heat stress to act as cytoprotective measures against excess superoxide formation [69]. An increase in oxidative stress may be expressed by the elevation in plasma levels of lipid peroxidation products (including malondialdehyde [70]. Increased activity of antioxidant enzymes have been associated with decreased MDA levels [71]. Curcumin was demonstrated to improve antioxidant status in heat-stressed hens by lowering serum MDA levels while promoting enzymatic activities of SOD and GSH-Px [46]. Our study revealed that MDA levels were reduced, whereas anti-oxidants such as CAT, SOD, and T-AOC were elevated in laying hens fed L-Cit. This implies that dietary L-Cit initiated antioxidant defenses to combat the excessive production of ROS. It had been reported that L-Cit can inhibit ROS formation through direct scavenging of hydroxyl radicals and/or chelation of copper to inhibit hydroxyl radicals production [72].

L-Cit inhibited GSH-Px activity in a dose-specific manner. This may result from L-Cit elevation of NO_x_ concentrations, improving the reaction between NO and oxygen to yield oxidant metabolites of NO which can suppress GSH-Px activity [73]. The GSH-Px enzyme belongs to the family of selenoproteins, as such carries a selenocysteine at its active site [74]. Research has shown that S-nitroso-N-acetyl-*D,* *L*-penicillamine (SNAP), an NO donor can irreversibly inactivate GSH-Px enzyme, since the NO released acts to modify the cysteine-like active site on GSH-Px [75]. Similarly, administration of peroxynitrite precursor, 3-morpholinosydnonimine-N-ethylcarbamide, as well as synthetic peroxynitrite inactivated bovine GSH-Px activity [76]. The implication for increasing NO availability is the direct attenuation of GSH-Px activity which may result in increased intracellular peroxides. However, since L-citrulline initiates the activity of SOD and CAT which also actively catalyze peroxides, we speculate that this would achieve a significant balance between oxidants and antioxidants for cellular homeostasis. Anti-oxidant enzymes interact in concert with each other, such that SOD catalytic product of hydrogen peroxide from superoxide ions is eliminated by CAT or GSH-Px activity [69].

Research has also linked L-Cit induction of NO production alongside with its anti-oxidant ability. L-Cit directly attenuated ROS production by downregulating the protein expression levels of p67phox, a critical component for NADH/NADPH superoxide generation [77]. *In vitro* studies using asymmetric dimethylarginine (ADMA), a non-selective NOS inhibitor, demonstrated L-Cit effectiveness to restore NO production and attenuate nitrosative stress [78]. Also, in atherosclerotic rabbits fed orally with L-arginine + L-citrulline alone or with antioxidants, there was a reduction in superoxide production, downregulation of oxidation-sensitive (Elk-1 and p-CREB) genes, with associated increments in NO synthase (eNOS) expressions and NO_x_ plasma concentrations [60].

## Conclusion

To our knowledge, this is the first study to report L-Cit induced modulation of arginine metabolism, nitric oxide synthesis, and antioxidant properties in chickens. Citrulline addition to diets, although having insignificant effects on production performance of laying hens, was able to induce the activities NOS isoforms (tNOS and iNOS) to initiate NO production. This study reveals that dietary citrulline supplementation can modulate the systemic availability of free amino acids, including arginine, citrulline, and ornithine, which are necessary for NO production. Furthermore, the anti-oxidant status was enhanced, following citrulline’s ability to reduce lipid peroxidation, while increasing the activities of antioxidants (SOD, CAT, and T-AOC). Therefore, the application of L-citrulline in diet formulation would enrich feeds with amino acids, antioxidants, and promote systemic NO production.

## Supplementary information

**Additional file 1: Fig. S1.** **Effect of different L-Cit supplementation levels on weekly rectal temperatures of laying hens.** Values are means ± SEM. (*n* = 12 birds).

**Additional file 2: Fig. S2.** **Effect of different L-supplementation levels on weekly production performance of laying hens.** (A) Egg weights (B) Laying rates and (C) Average daily feed intake of laying. Values are means ± SEM. Significantly different mean values are represented as *P < 0.05 (*n* = 12 birds per replicate).

**Additional file 3: Table S1.** Linear regression model for laying rate as a function of dietary citrulline levels in laying hens at 34–42 weeks old.

**Additional file 4: Table S2.** Quadratic regression model for laying rate as a function of dietary citrulline levels in laying hens at 34–42 weeks old.

## Data Availability

Datasets obtained and analyzed in this research are included within this article (and the supplementary data files).
