# Supplementary material for: Dietary L-citrulline supplementation modulates nitric oxide synthesis and anti-oxidant status of laying hens during summer season
Source: J Anim Sci Biotechnol. 2020 Oct 12;11:103. doi: 10.1186/s40104-020-00507-5 (PMC7549236; doi:10.1186/s40104-020-00507-5)
Supplement: Supplementary file 1 — Additional file 1: Fig. S1. Effect of different L-Cit supplementation levels on weekly rectal temperatures of laying hens. Values are means ± SEM. (n = 12 birds). [file 40104_2020_507_MOESM1_ESM.docx]

**Fig. S1. Effect of different L-Cit supplementation levels on weekly rectal temperatures of laying hens.** Values are means ± SEM. (*n*= 12 birds)
