# Supplementary material for: Dietary L-citrulline supplementation modulates nitric oxide synthesis and anti-oxidant status of laying hens during summer season
Source: J Anim Sci Biotechnol. 2020 Oct 12;11:103. doi: 10.1186/s40104-020-00507-5 (PMC7549236; doi:10.1186/s40104-020-00507-5)
Supplement: Supplementary file 2 — Additional file 2: Fig. S2. Effect of different L-supplementation levels on weekly production performance of laying hens. (A) Egg weights (B) Laying rates and (C) Average daily feed intake of laying. Values are means ± SEM. Significantly different mean values are represented as *P < 0.05 (n = 12 birds per replicate). [file 40104_2020_507_MOESM2_ESM.docx]

**Fig. S2. Effect of different L-supplementation levels on weekly production performance of laying hens.** (A) Egg weights (B) Laying rates and (C) Average daily feed intake of laying. Values are means ± SEM. Significantly different mean values are represented as **P* < 0.05 (*n*=12 birds per replicate)
