# Supplementary material for: Dietary L-citrulline supplementation modulates nitric oxide synthesis and anti-oxidant status of laying hens during summer season
Source: J Anim Sci Biotechnol. 2020 Oct 12;11:103. doi: 10.1186/s40104-020-00507-5 (PMC7549236; doi:10.1186/s40104-020-00507-5)
Supplement: Supplementary file 3 — Additional file 3: Table S1. Linear regression model for laying rate as a function of dietary citrulline levels in laying hens at 34–42 weeks old. [file 40104_2020_507_MOESM3_ESM.docx]

**Table S1.** Linear regression model for laying rate as a function of dietary citrulline levels in laying hens at 34-42 weeks old

| Variables | F-value | *R*^2^ | Estimate | Std error | *t* value | *P*-value |
| --- | --- | --- | --- | --- | --- | --- |
| Intercept |  |  | 82.859 | 2.149 | 38.564 | 0.001*** |
| Citrulline | (1, 22) 1.485 | 0.063 | ﹣4.571 | 3.751 | ﹣1.219 | 0.236 |
