# Supplementary material for: Dietary L-citrulline supplementation modulates nitric oxide synthesis and anti-oxidant status of laying hens during summer season
Source: J Anim Sci Biotechnol. 2020 Oct 12;11:103. doi: 10.1186/s40104-020-00507-5 (PMC7549236; doi:10.1186/s40104-020-00507-5)
Supplement: Supplementary file 4 — Additional file 4: Table S2. Quadratic regression model for laying rate as a function of dietary citrulline levels in laying hens at 34–42 weeks old. [file 40104_2020_507_MOESM4_ESM.docx]

**Table S2.** Quadratic regression model for laying rate as a function of dietary citrulline levels in laying hens at 34-42 weeks old

| Variables | F-value | *R*^2^ | Estimate | Std error | *t* value | *P*-value |
| --- | --- | --- | --- | --- | --- | --- |
| Intercept |  |  | 82.605 | 2.719 | 30.382 | 0.001*** |
| Citrulline |  |  | ﹣2.468 | 13.805 | ﹣0.176 | 0.860 |
| Citrulline (squared) | (2, 21) 0.722 | 0.064 | ﹣2.030 | 12.804 | ﹣0.159 | 0.876 |
